# Portal dosimetry in radiotherapy repeatability evaluation

**DOI:** 10.1002/acm2.13123

**Published:** 2020-12-12

**Authors:** Krzysztof Ślosarek, Dominika Plaza, Aleksandra Nas, Marta Reudelsdorf, Jacek Wendykier, Barbara Bekman, Aleksandra Grządziel

**Affiliations:** ^1^ Radiotherapy Planning Department Maria Skłodowska‐Curie National Research Institute of Oncology Gliwice Branch Poland

**Keywords:** EPID, fluence maps, gamma analysis, transit dosimetry

## Abstract

The accuracy of radiotherapy is the subject of continuous discussion, and dosimetry methods, particularly in dynamic techniques, are being developed. At the same time, many oncology centers develop quality procedures, including pretreatment and online dose verification and proper patient tracking methods. This work aims to present the possibility of using portal dosimetry in the assessment of radiotherapy repeatability. The analysis was conducted on 74 cases treated with dynamic techniques. Transit dosimetry was made for each collision‐free radiation beam. It allowed the comparison of summary fluence maps, obtained for fractions with the corresponding summary maps from all other treatment fractions. For evaluation of the compatibility in the fluence map pairs (6798), the gamma coefficient was calculated. The results were considered in four groups, depending on the used radiotherapy technique: stereotactic fractionated radiotherapy, breath‐hold, free‐breathing, and conventionally fractionated other cases. The *chi^2^* or *Fisher's exact* test was made depending on the size of the analyzed set and also *Mann–Whitney U‐test* was used to compare treatment repeatability of different techniques. The aim was to test whether the null hypothesis of error‐free therapy was met. The patient is treated repeatedly if the *P*‐value in all the fluence maps sets is higher than the level of 0.01. The best compatibility between treatment fractions was obtained for the stereotactic technique. The technique with breath‐holding gave the lowest percentage of compliance of the analyzed fluence pairs. The results indicate that the repeatability of the treatment is associated with the radiotherapy technique. Treated volume location is also an essential factor found in the evaluation of treatment accuracy. The EPID device is a useful tool in assessing the repeatability of radiotherapy. The proposed method of fluence maps comparison also allows us to assess in which therapeutic session the patient was treated differently from the other fractions.

## 1 INTRODUCTION

Radiotherapy, as a treatment using ionizing radiation, is an effective method of oncology. However, several criteria must be met. One of them is the precision of the radiation dose delivery.

**FIG. 1:**
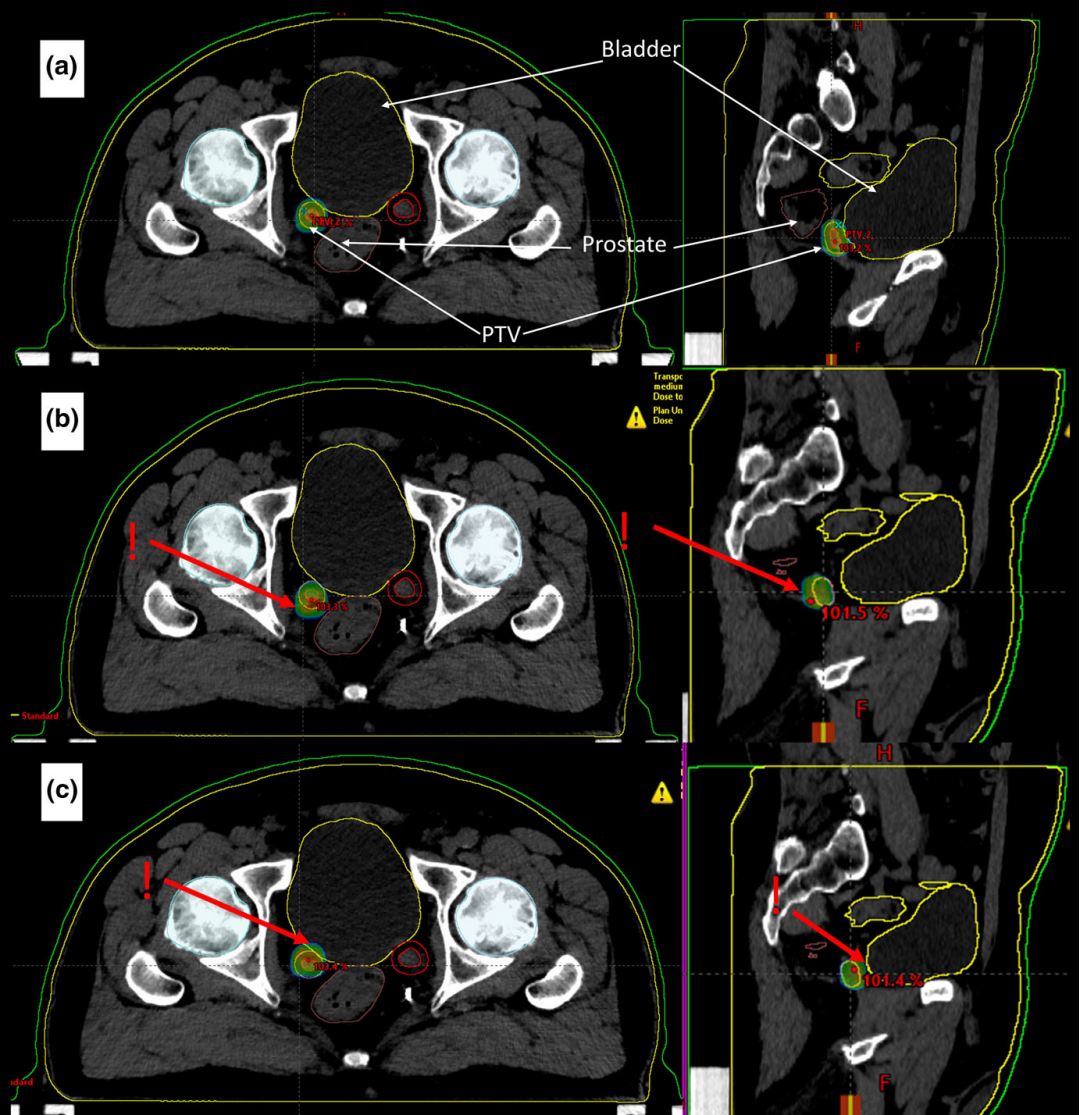
Example of planned and changed dose distribution in lymph node radiotherapy for the VMAT technique with 6 MV FFF beam. Visible contours of PTV — lymph node with margin (red) and critical structures: bladder (yellow), rectum (brown), femoral heads (blue). Dose distribution ≥90% of the planned total dose; (a) in the planned patient treatment position. (b) after moving the patient 3mm forward in the anteroposterior direction relative to the planned position (c) after moving the patient 3mm backward relative to the planned position.

One of the principles in medicine is "first, do no harm." This dictum applied in radiotherapy means to protect the critical organs as much as possible. Dynamic intensity‐modulated radiotherapy (IMRT) or volumetric modulated arc therapy (VMAT) techniques allow to achieve a dose distribution that meets the physician's expectations and indications of radiotherapeutic protocols. Therapeutic doses in the treated volumes are achieved while doses in critical organs located in the immediate vicinity are acceptable. Unfortunately, any change in the geometry of the irradiated volume, or an inaccurate positioning of the patient, result in a significant change in the dose distributions. Figure 1 shows the change in dose distribution in the target area and critical organs once the patient treatment position has changed against the planned position. Simulation of possible changes in dose distributions can be done during the treatment planning in most of the modern treatment planning systems. This option allows us to assess how dose changes in target volume and critical organs affect the likelihood of local control and typical tissue complications.

Figure 1(b) presents dose delivered to the bladder and rectum which is lower than the planned one. This situation is not clinically relevant. Unfortunately, at the same time the dose in the PTV volume is lower than planned, which can result in lower local control probability. Figure 1(c) presents irradiation of critical structures with a higher dose. This can lead to complications after the treatment.

In radiotherapy, orthogonal x‐ray imaging or cone‐beam tomography (CBCT) is performed before the therapeutic session to minimize the differences between the planned and actual patient position. The acquired two‐ or three‐dimensional images are compared with reference images sent from the treatment planning system. Nevertheless, one should remember that on C‐arm linear accelerators, the image verifications are mostly performed before switching to radiation exposure. If there are differences between the planned and the actual patient position, the action is required. Patient position is adjusted to the planned one. However, during the therapeutic session, the patient may intentionally or passively change the position. In such case, the actual dose distribution in the patient body is unknown. Hence, it seems so important to control these changes occurring during the therapeutic session.

Modern C‐arm accelerators are equipped with real‐time dose‐monitoring systems, that is, portal matrices (EPID — Electronic Portal Imaging Device). Therefore, in addition to the old‐fashioned use of matrices to control the patient treatment position and regular use for pretreatment dosimetry, EPID matrices allow to register a signal that can later be reconstructed, as a dose distribution during the therapeutic session with the patient.^1^, ^2^, ^3^, ^4^, ^5^, ^6^ The signal measured by the EPID matrix is called the fluence map.

For physics and computer science, the VMAT technique with an acting EPID is a type of megavolt cone‐beam computed tomography. From that, as in CT, the needful information is given to reconstruct the patient anatomy and dose distribution. There are many published articles on how to calculate the dose distribution based on the real‐time EPID image.^7^, ^8^, ^9^, ^10^ However, the software described in the literature is mostly not commercial. If it is available, its use is time‐consuming and difficult in clinical practice. Nowadays, the compatibility of dose distribution between the calculations from treatment planning system (TPS) and measured dose distribution in dynamic techniques is performed without a patient.

Since useful tools to compare the measured fluence map with that calculated during treatment planning are available, one can focus solely on treatment repeatability. Fluence maps measured during the therapeutic session can be compared with each other. It can be assumed that the patient is repeatably treated if these do not differ from each other. A patient who is treated repeatably is highly likely to be treated as planned. Measurement of a dose or fluence map, taking into account the patient's body, is called transit dosimetry.^11^

**FIG. 2:**
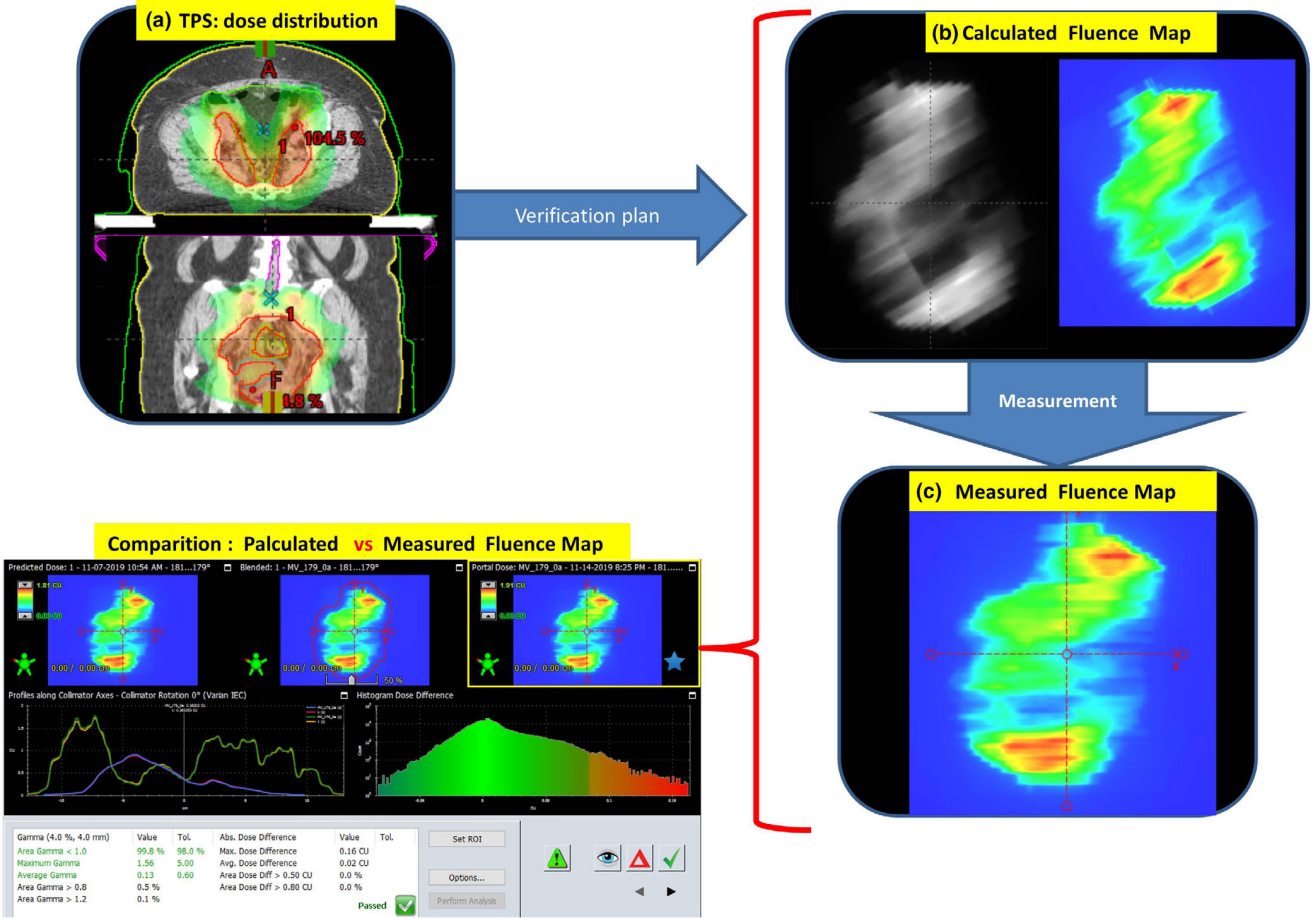
Procedure for generating, measuring and comparing fluence maps. The treatment planning system calculates the dose distribution in the patient's body (a). Then it is converted maintaining all the parameters of the beams into a verification plan and reference fluence maps are generated. (b). Fluence maps are measured using the EPID matrix (c). In the next step, the calculated fluence map (b) is compared with the measured one (c). If gamma is smaller than 1 in 98% analyzed points, comparisons: calculated vs. measured fluence maps — pass.

The aforementioned common use of portal matrices allows to check the dose distribution by measuring fluence maps before the therapeutic session without the patient.^12^, ^13^, ^14^ Beforehand, based on the patient planned dose distribution, the fluence maps are generated in the treatment planning system for all fields (Calculated fluence map). They are reference maps for those measured later on the treatment unit (Measured fluence map) (Fig. 2).

The process of pretreatment verification with patient absence is performed to check whether the movement of the collimator leaves and the gantry can be carried out, in other words, if the treatment is possible from the strictly technical point of view. The calculated dose distribution in a patient is converted into a fluence map for the patient geometry conditions. The compatibility of both types of fluence maps gives certainty that the radiation registered by EPID is generated correctly without a patient being present. Dose verification is more complicated. It is assumed that if there is an agreement between the calculated and measured fluence maps, there is an agreement between the calculated and realized dose distribution. It can be assumed that if the additional condition of correct positioning of the patient and maintaining this position during the entire irradiation is met.

**FIG. 3:**
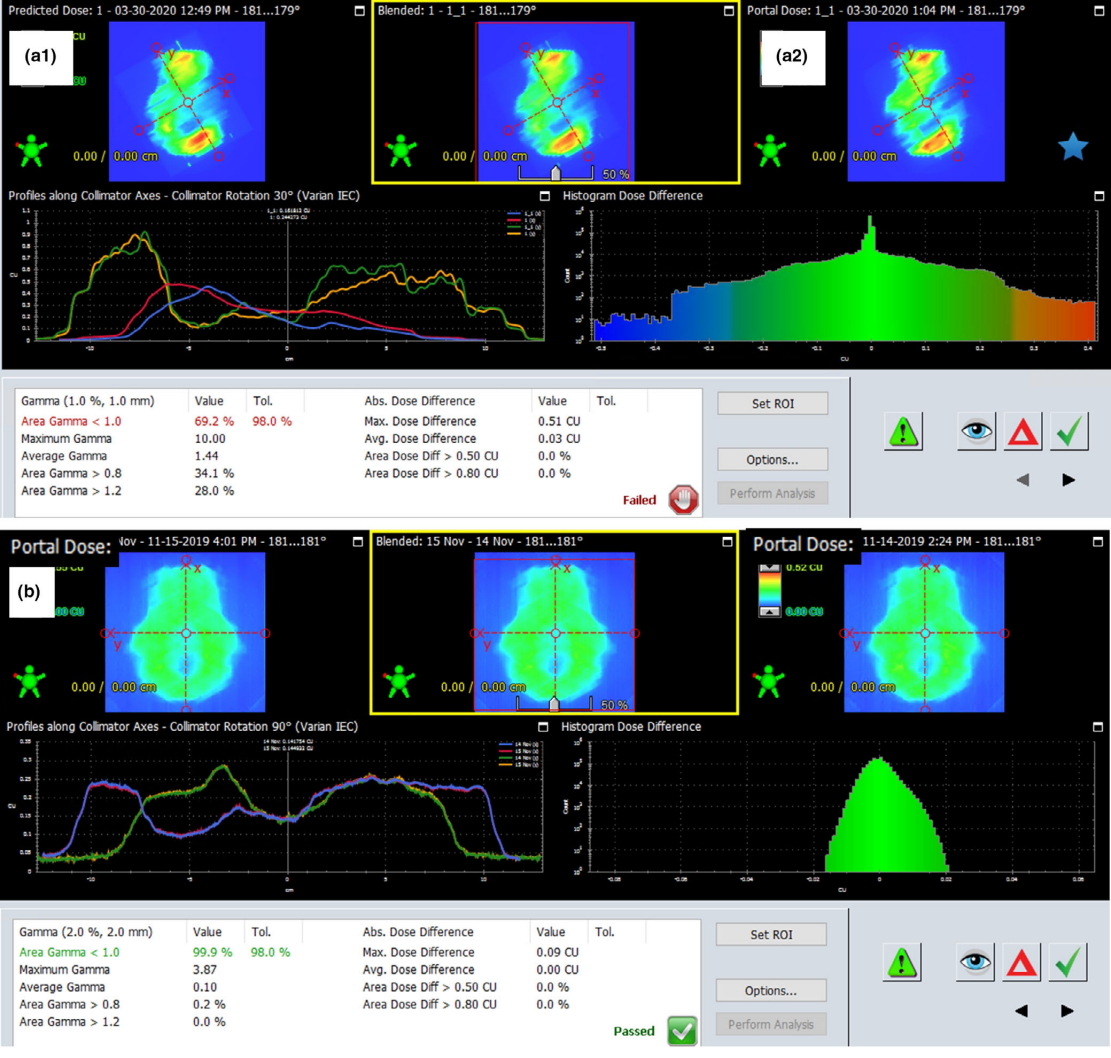
Application of the gamma coefficient. (a) comparison of two calculated fluence maps, A1 field with movable collimator jaws, A2 field with fixed jaw positions. The analysis shows that the state of the jaws has an impact on a fluence map. (b) Comparison of fluence maps measured in the patient's body during the therapeutic session in 2 days of treatment.

The parameter adopted for comparing two data sets, including dose distributions or fluence maps, is the gamma coefficient.^15^ Each measurement is characterized by uncertainty influenced by several factors, such as the precision of the measurement system settings. The gamma coefficient defined in the form of values given in brackets (ΔD = x %, DTA = y mm, 98%) means that the acceptable agreement between two data sets is x %, their offset (DTA) is y mm in 98% of analyzed field points. If these conditions are met, then the gamma value is less than or equal to 1. This method of assessing the compliance of two quantities can be used to compare calculations with measurements (Fig. 2) as a mandatory QA procedure in radiotherapy to compare two calculated data (e.g., a good option for testing calculation algorithms) and to compare two measurement data (Fig. 3).

Figure 2 presents the algorithm of dose distribution verification as the calculated and measured fluence maps comparison. It is the classic QA process. Figure 3 shows the possibility of comparing two fluence maps measured during the therapeutic session. The experience described in the paper of Klimas et al.^16^ shows that the repeatability of fluence maps measurements is 2%, 2 mm in 98% of analyzed field points. Therefore, EPID can be used to verify differences between fluence maps. The given compliance criterion, developed for the phantom conditions, was used in this study as the starting point for fluence maps comparison in clinical cases.

The study aims to present the possibilities of EPID dosimetry use in assessing the repeatability of radiotherapy.

### 1.A MATERIALS AND METHODS

During radiotherapeutic sessions, transit dosimetry was made for each collision‐free radiation beam. It means that the EPID matrix measured the fluence maps while the beams passed through the patient body. All measurements were performed on a‐Si 1200 imagers installed on TrueBeam and Edge accelerators (Varian Medical Systems, Palo Alto, CA, USA). The arrays have an active area of 40 cm x 40 cm with a pixel number of 1190 x 1190 and a pixel resolution of 0.34 mm at isocenter.^17^ In the studied cases, 6 MV, 15 MV, 6 MV FFF, and 10 MV FFF photon beams were used in dynamic plans. Two types of dynamic plans with intensity modulation were used: IMRT and VMAT. If the treatment plan consisted of several fields or arcs, all measured fluence maps were added together, and a summary fluence map was generated for a given radiation fraction. For this purpose, the *Composite Image* function was used in the *Portal Dosimetry* module of the ARIA v 15.1 software (Varian Medical Systems, Palo Alto, CA USA). Summary fluence maps obtained for a single fraction were compared with the corresponding summary maps from all other treatment fractions. All pairs of fluence maps were used for the analysis. The similarity in fluence maps pairs was assessed based on the gamma coefficient calculated in the analyzed field. The field bounded by the multileaf collimator (MLC) enlarged by 1cm was taken for evaluation.

Acceptable dose difference values of 2% and dose shift value by 2 mm were assumed. Pairs of fluence maps were agreed if this criteria (γ[2%,2 mm]<1), are met in 98% of analyzed field points. The adopted gamma criteria: (2%, 2 mm, 98%). The *Portal Dosimetry* module was also used to compare fluence maps and gamma calculations.^18^ A situation in which all pairs of fluence maps met gamma criteria without exception was considered a fully repeatable irradiation of the patient. Fluence maps measurements and analysis were carried out for 74 patients who underwent one of the following four radiation therapies: Fractionated Stereotactic Radiation Therapy (FSRT) in the brain, head and neck, and prostate region; breath‐hold (BH) in breast cases; free‐breathing (FB) in the chest region and other cases (DC), that is, irradiation of the mediastinum, abdomen, and pelvis with conventional fractionation. In the BH and FB, the respiratory gating system was used. In total, 6798 fluence maps comparisons were made. For each patient, all fractions were compared. All fractions were compared to one another, as it is impossible to indicate which measurement is the reference value. This procedure showed how many analyzed pairs of fluence maps are compatible and how many do not meet the defined criteria of the gamma coefficient.

The null hypothesis (H_0_) assumes that treatment is repeatable when all pairs of fluence maps match, making all comparisons meet the gamma criteria. The alternative H_1_ hypothesis is: the treatment is not reproducible due to the lack of agreement between the compared fluence maps. The assumption is that the patient is treated in an unrepeatable way if the *p‐value* between the compared sets is lower than 0.01. There is no base to reject the null hypothesis. The *chi^2^* or *Fisher's exact* test was made depending on the analyzed set size.^19^ The *Mann–Whitney U‐test* was used to compare the treatment repeatability of different irradiation techniques. All tests were two‐tailed with type I error rate fixed at 0.01. Statistical analyzes were performed with Statistica v 12 software (StatSoft, Inc., 1984–2014). In Table 1, a C value equal to the number of all fluence maps comparisons and a D value equal to zero represents the null hypothesis.

**TABLE 1:** Possible results of analysis of fluence maps pairs.

	Number of matching comparisons	Number of incompatible comparisons	Number of all comparisons
Analyzed RT	A	B	A + B
Theoretical RT	C	D	C + D

If we consider ten fractions of radiation therapy and EPID measurement is performed each day, we obtain a set of 45 comparisons of fluence maps. Assuming that radiation therapy is performed in a repeatable way, then C = 45 and D = 0. If 44 comparisons meet the gamma criteria (A = 44), and one comparison does not (B = 1), then the *chi^2^* test shows that *P* = 0.3146. There is no base to reject the null hypothesis. It means that radiotherapy is repeatable if incompatibility occurs in one comparison only. However, if six compared fluence maps do not meet the gamma criteria (B = 6), and 39 meet (A = 39), the *chi^2^* test shows value of *P* = 0.0059. Consequently, one can reject the null hypothesis and accept an alternative: the patient is not being treated in a repeated way.

This way of comparing fluence maps also allows us to assess in which therapeutic session the patient was treated differently from the other fractions.

## 2 RESULTS

The performed analysis indicates that the highest percentage of repeatable treatment is obtained in stereotactic radiotherapy and the lowest in the BH technique (Table 2). It means that statistical significance (*P*‐value higher than 0.01) estimated in *chi^2^* or *Fisher's exact* test shows the compliance with measured fluence maps for given gamma criteria of (2%, 2 mm, 98%).

**TABLE 2:** Summary results of fluence maps pairs analysis for different irradiation techniques.

RT technique	Number of patients (∑ = 74)	Number of analyzed fluence maps pairs (∑ = 6798)	Gamma (2%,2mm,98%) < 1	
			Average percentage of reproducible fractions [%]	ch^2^ test *P*‐value
FSRT	23	124	90.58	0.997
DC	20	1846	75.37	< 0.01
FB	11	1900	57.87	< 0.01
BH	20	2928	20.70	< 0.01

FSRT, Fractionated Stereotactic Radiation Therapy; DC, chest region; FB, free‐breathing; BH, breath hold.

*P‐values* presented in Table 2 indicate that all FSRT patients were treated repeatedly. However, in two cases of abdominal nodes, despite statistically repeatable treatment, in three of six fractions, the difference in the dose was higher than 2%. The place of its deposition differed from the planned one by more than 2 mm. Except these two cases, 98% of the analyzed fluence maps pairs meet the gamma criterion.

**FIG. 4:**
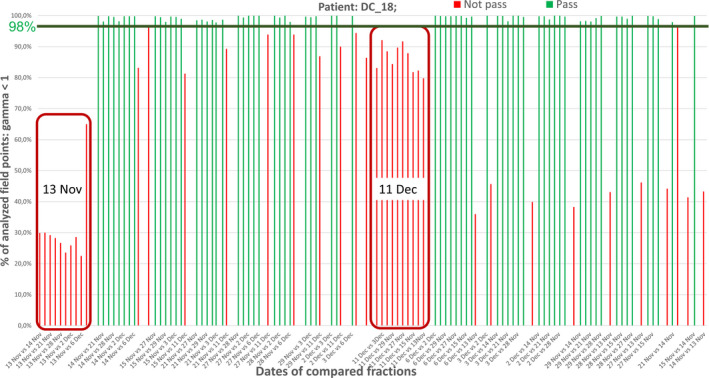
Comparisons of measured fluence maps between different fractions show, that on November 13 and December 11, patient was treated differently as compared to other days. On November 13 only 30%, and on December 11%–80% of analyzed field points meet gamma criterion.

The results of the analysis performed for the DC group in the mediastinum, abdominal cavity, and pelvis with conventional fractionation demonstrate that for an average of 75% of the fluence maps pairs, the gamma coefficient is less than or equal to one. The statistical analysis shows that the patients in this group are not irradiated in a repeatable way. Since it is a rectal treatment, significant changes in anatomic shape and volume of patient's organs are likely to affect the result achieved. Additionally, there is one patient in this group, who is irradiated in two fractions that differ considerably from other fractions. The analysis presented in Fig. 4 shows that on November 13 and December 11, the compatibility of the compared fluence maps with other maps was much worse.

In the FB techniques for 58% of pairs, the gamma criteria are met (Table 2). A statistical test indicates that no patient was irradiated repeatedly. For all cases, the *p‐value* is less than 0.01.

The analysis of the fluence maps pairs in BH cases showed that the average compatibility of the analyzed fluence pairs is 20% (Table 2). From a statistical point of view, no patient in BH group was irradiated repeatedly. For all cases, the *P‐value* is less than 0.01. In the chest wall area, the 2 mm shifts are unavoidable. The radiation dose is related to the energy deposited in the matter. Therefore, the lung volume changes due to respiratory movements affect the dose distribution and the measured fluence map.

The statistical analysis done for comparison of FSRT, DC, FB, and HB techniques shows that there are significant differences between these groups. The *P*‐values in Mann–Whitney U‐ test are as follows: for FSRT vs. BH, DC vs. BH, and FB vs. BH are lower than 0.01. It means that the BH technique differs significantly from the others concerning the repeatability of the fractions.

## 3 DISCUSSION

**FIG. 5:**
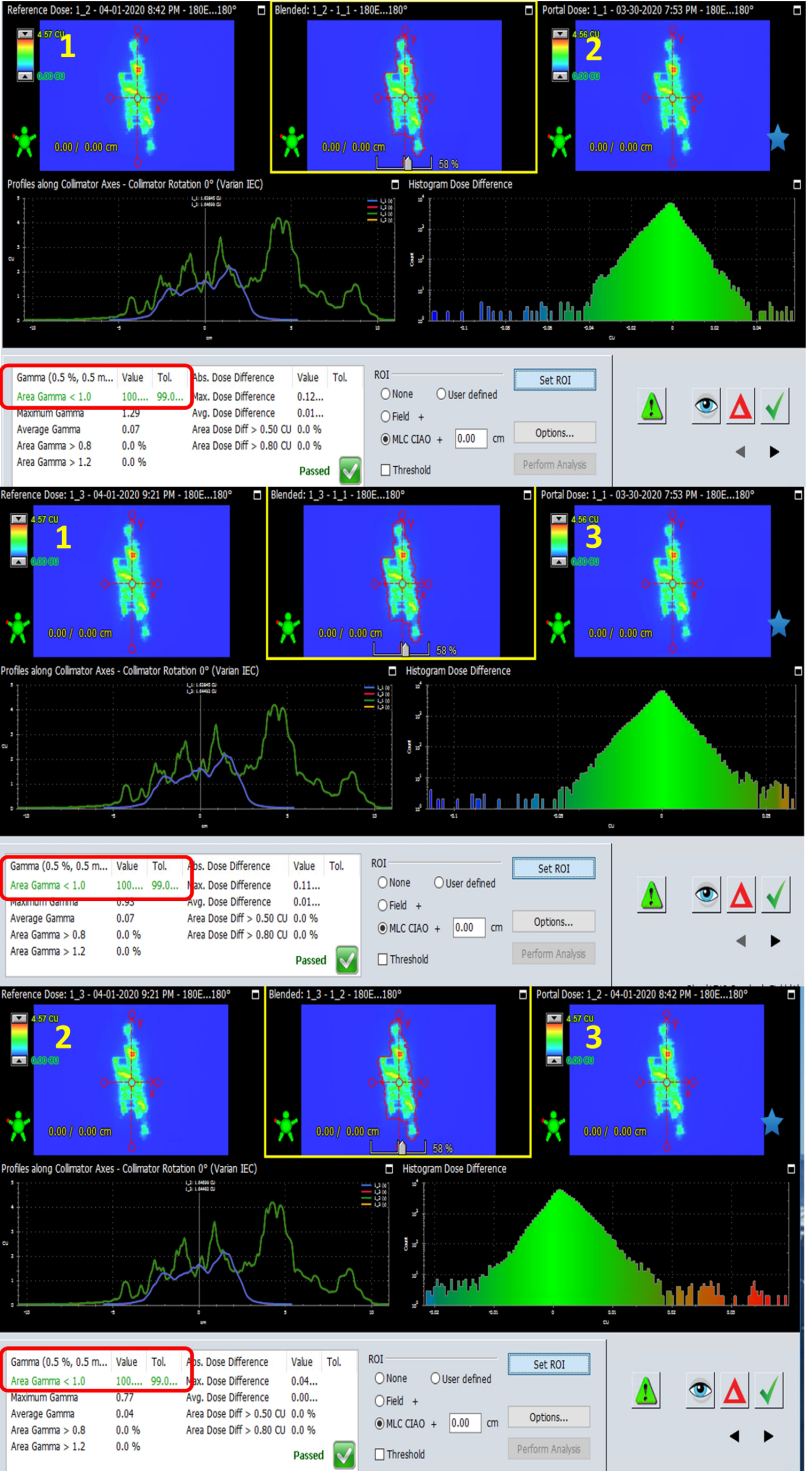
Fluence maps measured three times.

Comparing the fluence maps measurements in the absence of the patient is the standard QA procedure for radiotherapy in dynamic techniques. Figure 5 shows the results of comparing three pretreatment fluence map measurements.

Comparison was performed on first vs. second fraction, first vs. third fraction, and second vs. third fraction. The gamma coefficient for 0.5% and 0.5mm is less than or equal to 1 in 100% of the analyzed field. This means that the repeatability of measurements (EPID) is much lower than 2% and 2mm. It can be assumed that the differences between measurements higher than these values are related to the mobility of the patient.

Previous experiments indicate that such a high degree of maps similarity for (0.5%, 0.5 mm, 99%) gamma criteria are not always obtained and are challenging to achieve.^16^ Therefore, the values of (2%, 2 mm, 98%) were adopted. They are sufficient to assure the accuracy of measurement system. Higher differences can be caused by the presence of patient between the radiation source and measurement system.

It can be concluded that only these patients, who are treated with the fractionated stereotactic technique, are irradiated repeatably. The presented results indicate that the repeatability of the treatment is associated with the radiotherapy technique. It is not surprising that the stereotactic technique is performed with high precision and repeatability. All stereotactic patients included in the analysis were treated in this way. It is definitely due to the number of fractions and the excellent immobilization of the patient. Locations of the tumors in stereotactic treatment are crucial too. In the brain region, changes in the position of the anatomical structures are minimal. In extracranial cases, anatomy changes are observed more frequently. This was confirmed based on the results from two cases — when the lymph nodes located in the abdominal cavity were irradiated. The question is what difference in the dose and its shift have to be assumed in the gamma coefficient calculations, to proof the lack of treatment repeatability, using real‐time fluence map measurements? There is no doubt that they should be associated with the location of the tumor. For brain and craniofacial tumors, a tolerance of 2% and 2 mm is optimal. The analysis shows that all patients in this group were treated reproducibly. In the mediastinum and pelvis tumor location (DC group), the analysis shows that no patient was irradiated repeatably for the (2%, 2 mm, 98%) conditions. Shifts in anatomical organs position in the abdominal cavity are undoubtedly higher than 2 mm, so the values of 3% and 3 mm were adopted, and the analysis was performed again. The reanalysis in DC group shows that approximately 88% of the analyzed pairs of fluence maps meet the gamma condition of (3%, 3 mm, 98%). The value of *P* = 0.0147 means that there are no differences between the theoretical and analyzed groups. The patients were treated in the repeatable way.

In the FB and BH groups, very little compatibility between pairs of fluence maps was obtained. Therefore the analysis was redone for 3% and 5 mm conditions, which allowed to achieve 98% of the analyzed surface. In irradiation of chest wall after mastectomy at FB, assuming new gamma criteria, 94% of patients were treated repeatedly (*P* = 0.018), except two patients, whose results differed considerably from the rest. Therefore, it can be assumed that patients treated with this technique are irradiated repeatedly within the dose range of 3% and 5mm shift.

In the group of breast cancer patients irradiated with VMAT on BH technique, the reanalysis was performed for (4%, 5 mm, 98%) condition. Only 75% of the analyzed pairs of fluence maps meet this criterion, and the *P*‐value lower than 0.01 indicates that the patients were treated in a repeated manner. For this technique further analysis should be performed in order to find out whether the value of dose offset should be enlarged to 6–7 mm or the surface percentage should be reduced to, for example, 95%.

Resource data confirm that the use of EPID in radiotherapy verification reduces errors, which undoubtedly improves treatment results.^20^, ^21^, ^22^, ^23^ Transit dosimetry with EPID matrices can be used based on two methods — comparing calculated fluence maps with measured ones or by comparing all measured fluence maps with one another. We chose the latter, and we believe it the right one to assess the repeatability.

## 4 CONCLUSIONS

1. The system which measures the fluence maps during the therapeutic session makes it possible to assess the treatment repeatability.
2. Analysis of fluence maps performed based on EPID, during the therapeutic session, shows significant differences in the dose deposited in patient body, depending on the applied radiotherapy technique and tumor location. The highest repeatability was achieved with the stereotactic techniques, whereas the lowest during breast radiation therapy.
3. The calculations show that various radiation techniques and various tumor locations require different criteria, in order to optimize the treatment repeatability assessment.
4. All additional actions aimed at improving the radiotherapy results and/or patient's safety is extremely important. Fluence maps assessment is an example of such action, especially given the fact that it does not extend the treatment duration, and does not expose patient to additional dose.
5. Further research is required in order to define the optimum repeatability criteria for various techniques and tumor locations.

## AUTHOR'S CONTRIBUTION

Krzysztof Ślosarek conceived and designed the analysis, contributed data or analysis tools, and wrote the paper.

Dominika Plaza, Aleksandra Nas, and Marta Reudelsdorf collected the data.

Jacek Wendykier and Barbara Bekman performed the analysis and other contribution: language corrections.

Aleksandra Grządziel performed the analysis, wrote the paper, and other contribution: language corrections.
